# A rare case of watery diarrhea, hypokalemia and achlorhydria syndrome caused by pheochromocytoma

**DOI:** 10.1186/1471-2407-14-553

**Published:** 2014-07-31

**Authors:** Jingjing Jiang, Li Zhang, Zhaodi Wu, Zhilong Ai, Yingyong Hou, Zhiqiang Lu, Xin Gao

**Affiliations:** Department of Endocrinology and Metabolism, Zhongshan Hospital, Fudan University, 180 Fenglin Road, Shanghai, 200032 P.R. China; Department of Urology, Zhongshan Hospital, Fudan University, 180 Fenglin Road, Shanghai, 200032 P.R. China; Department of Gastroenterology, Zhongshan Hospital, Fudan University, 180 Fenglin Road, Shanghai, 200032 P.R. China; Department of General Surgery, Zhongshan Hospital, Fudan University, 180 Fenglin Road, Shanghai, 200032 P.R. China; Department of Pathology, Zhongshan Hospital, Fudan University, 180 Fenglin Road, Shanghai, 200032 P.R. China

**Keywords:** Vasoactive intestinal polypeptide, Pheochromocytoma, Hypercalcemia, Bone metabolism

## Abstract

**Background:**

A rare syndrome of watery diarrhea, hypokalemia and achlorhydria (WDHA) is usually caused by pancreatic endocrine tumors that secrete excessive vasoactive intestinal polypeptide (VIP). Here we report a rare case of WDHA caused by a pheochromocytoma.

**Case presentation:**

A 45-year old male presented with persistent and progressive watery diarrhea for half a year, and was treated with dialysis due to azotemia, hypokalemia, hypercalcemia and metabolic acidosis. A right adrenal mass was found by ultrasonography, and Positron Emission Tomography-Computed Tomography (PET-CT) showed the tumor was hyper-metabolic. Levels of plasma normetanephrine (NMN) and serum chromogranin A (CgA) were significantly elevated. Immunohistochemistry analysis of the adrenal tumor was strongly positive for CgA, synaptophysin and VIP. The patient fully recovered from WDHA syndrome soon after surgery, as reflected in that diarrhea stopped, levels of plasma NMN, serum CgA, and electrolytes returned to normal thus no dialysis was needed. The patient remained disease free in a 12-months follow-up period.

**Conclusion:**

We report an extremely rare case of pheochromocytoma causing WDHA syndrome and uremia, which the patient completely recovered from after tumor resection.

## Background

Vasoactive intestinal peptide (VIP) is a 28-amnio acid peptide that may cause secretory diarrhea when overproduced by activating adenylate cyclase. A rare syndrome of watery diarrhea associated with hypokalemia and achlorhydria (WDHA) due to hypersecretion of VIP was described initially by Verner and Morrison in 1958 [1]. This syndrome is usually associated with pancreatic endocrine tumors (VIPomas), with only a few exceptions. In this report, we describe a case of WDHA caused by a VIP-positive pheochromocytoma. Surgical resection of the tumor relieved all the symptoms and normalized all the relevant biochemical characteristics in the patient.

## Case presentation

A 45-year old man presented with persistent and progressive watery diarrhea for half a year. He was initially admitted to a local hospital because he suddenly lost consciousness, during which his blood pressure was unmeasurable. Emergency lab tests revealed elevated white blood cell count (WBC 21.4 × 10^9^/L, N 86.4%), hypercreatinemia (Cr 647umol/L) and hypokalemia (K 2.9 mmol/L). Arterial blood gas tests indicated metabolic acidosis and hypoxia (pH 7.16, HCO_3_ 10 mmol/L, PO_2_ 70%). He was intubated, maintained on hemodialysis and treated with fluid and antibiotics intravenously. After his condition improved, he was transferred to our hospital for further diagnosis and treatment.

**Figure 1:**
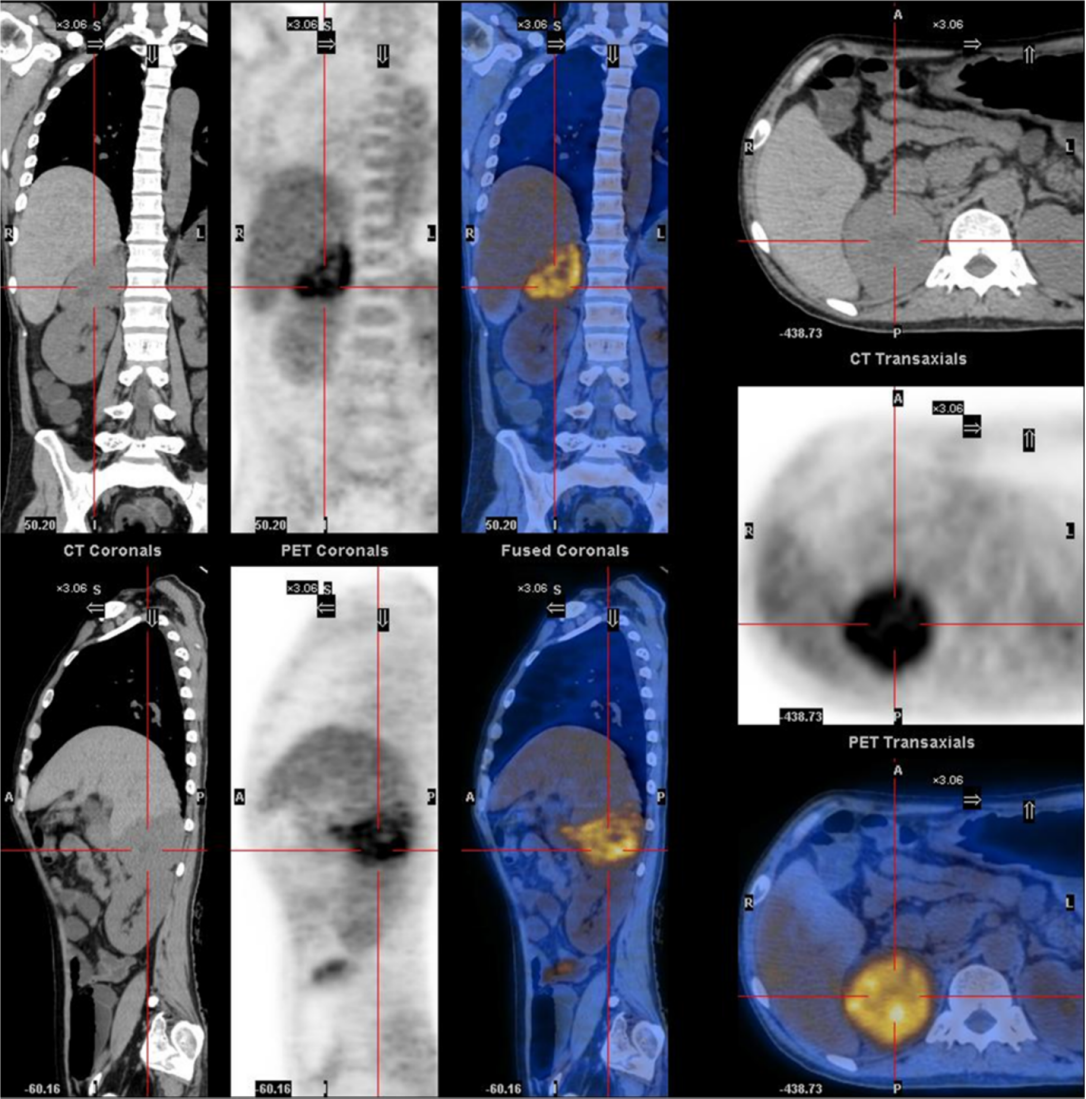
**A PET-CT scan demonstrating a large, well defined adrenal mass.** A large hyper-metabolic mass was located at the right adrenal region. The hypodense areas at the center indicated necrosis. Massive fluid collection could be found in the colon.

On admission, his blood pressure (BP) was around 90/55 mmHg, and heart rate (HR) was around 100 bpm. The diarrhea and dehydration were so severe that daily intravenous infusion of 10000 ml fluid could barely maintain his BP and HR stable. Anuria persisted with an elevated level of serum Cr (Table 1). As such, the patient continued to receive regular dialysis, thrice a week. His diarrhea responded poorly to the treatment with diosmectite, loperamide or octreotide. Routine lab tests revealed severe hypokalemia and hypercalcemia (Table 1). Bone markers including osteocalcin, beta-CTX and P1NP were all elevated (Table 1), suggesting a high bone turnover rate. PTH was suppressed while calcitonin was elevated (Table 1). The elevated calcitonin lead to the identification of a thyroid nodule at the left lobe by ultrasound. An abdominal ultrasound identified a mass in the right adrenal region. Plasma Normetanephrine (NMN) and metanephrine (MN) were 9554.1 pg/ml and 169.1 pg/ml, respectively. Serum chromogranin A (CgA) was beyond the detection range (>740 ng/ml), and neuron specific enolase (NSE) normal (14.8 ng/ml). A PET-CT scan revealed a 7.5 × 6.1 cm hyper-metabolic adrenal mass, whose SUV_(max)_ was 5.41. Several hypodense areas showing no glucose metabolism were found at the central part of the mass, suggesting necrosis (Figure 1). A hypodense lesion, in size of 1.48 × 0.97 cm, was identified at the left lobe of the thyroid gland with a SUV_(max)_ of 2.4.

**Table 1:** **Follow up of electrolytes and hormones**

	Before surgery	After surgery	Six month later	Reference range
Cr (umol/L)	562	78	76	44-115
K + (mmol/L)	2.8	3.6	4.8	3.5-5.3
Ca2 + (mmol/L)	3.11	2.03	2.42	2.15-2.55
Albumin (g/L)	29	28	47	40-55
PTH (pg/ml)	8.7	52.5	55.3	15-65
Osteocalcin (ng/ml)	73.4	33.6	30.7	6-24.7
β-CTX (ng/ml)	>6.0	3.05	0.46	0.04-0.78
P1NP (ng/ml)	150.9	305.4	133.8	9.1-76.2
Calcitonin (pg/ml)	128.0	3.4	<2	<8.4
CgA (ng/ml)	>740	207.7	86.2	27-94
MN (pg/ml)	169.1	35.6	33.6	<96.6
NMN (pg/ml)	9554.1	79.6	51.1	<163

Serum electrolytes and hormones were measured before surgery, one week after surgery and six months later.

The patient underwent a combined surgery, which removed the adrenal mass and the left lobe of the thyroid gland simultaneously. The surgery was completed uneventfully. The patient’s BP and HR were stable during the resection of a 9 × 8 × 5 cm adrenal mass. The gross and microscopic appearance was the typical feature of pheochromocytoma, whereas the thyroid nodule appeared to be a benign adenoma. There was no evidence of medullary thyroid cancer.

After surgery, the patient’s symptoms relieved rapidly. Diarrhea stopped soon after surgery. Moreover, his daily urine increased gradually and serum Cr also lowered into a normal range without dialysis. Plasma NMN, serum PTH, calcitonin and electrolytes all were back to normal (Table 1). Osteocalcin and beta-CTX lowered significantly, while P1NP increased significantly (Table 1). Two weeks after surgery, the patient recovered completely and was discharged. Six months after surgery, a comprehensive follow-up check revealed no abnormalities in the relevant biochemical makers, except for a slightly high P1NP (Table 1). Till now, the patient has been followed for one year, showing no sign of recurrence.

### Discussion

The first case of pheochromocytoma that causes WDHA syndrome was described by Loehry in 1975 [2]. Based on histology, there are two subtypes for these VIP-secreting pheochromocytomas: the composite form (mixed pheochromocytoma and ganglioneuroma) and the classic form (pheochromocytoma only). The latter causing WDHA syndrome has been reported in only 11 cases to date [2–12], including the current one. The mean age of the 11 patients was 49 years old (ranging from 28 to 84 years). A preference for female was noted, since only 2 patients were men, including the current case. It is noteworthy that severe azotemia and uremia presented in our case have never been described in previous cases. Octreotide is a recommended medication for treatment of WDHA syndrome as it improves diarrhea in >75% of patients with VIPomas and reduces VIP secretion [13]. Despite being the recommended medication, VIP-secreting pheochromocytomas had a relatively poor response to octreotide, a finding consistent with previous reports, thus implying the lack of somatostatin receptors in these VIP-containing tumor cells [8].

**Figure 2:**
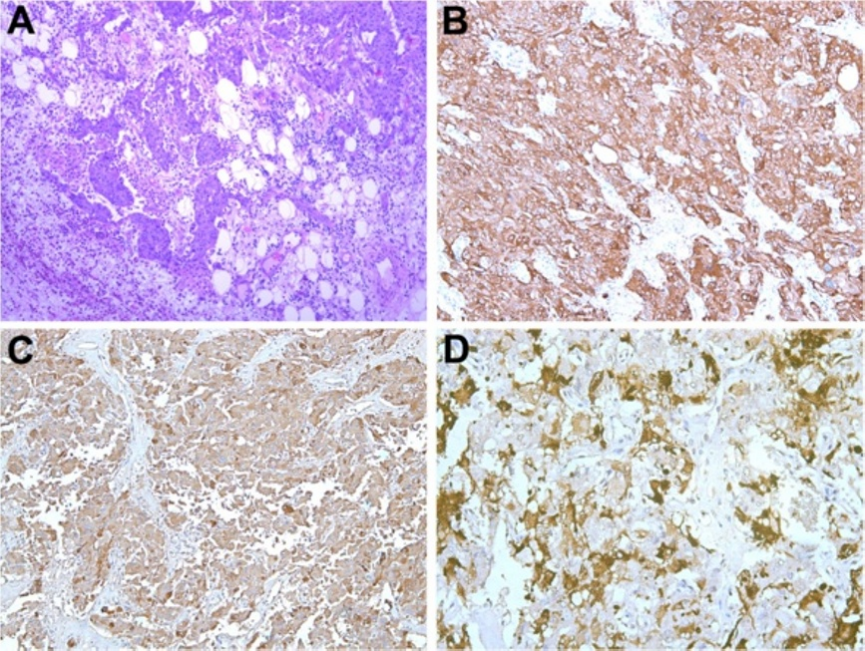
**Pathology slides of the resected specimen with different stains. A**: Hematoxylin and eosin stained section of the adrenal mass demonstrating adipose tissues infiltrated by tumor cells (×100). **B**: Immunohistochemical detection of synaptophysin in the tumor cells (×100). **C**: Immunohistochemical detection of chromogranin A in the tumor cells (×100). **D**: Immunohistochemical detection of VIP-positive cells (×200).

In the current case, the tumor appeared as a well-encapsuled brownish mass with a variegated cut surface and necrotic areas, showing a typical feature of pheochromocytoma. Histologically, adipose and fibrous tissues infiltrated by tumor cells were observed (Figure 2A). Almost all tumor cells (100%) were strongly positive for synaptophysin and CgA, and 40% of cells strongly positive for VIP. The VIP positive cells were clustered and scattered among the tumor cells (Figure 2B-D). Calcitonin staining was negative (data not shown). No ganglioneuroma component was identified, ruling out the composite form. According to the IHC staining, the current case can be diagnosed as a VIP-secreting pheochromocytoma. Unfortunately, this was not confirmed by biochemically measuring the secretion of VIP from the tumor, because the assay is currently unavailable in mainland China. It is noteworthy that no malignancy was described in the previous 10 cases. However, the present case showed infiltration of tumor cells into adipose tissue, large areas of necrosis in the tumor and intravascular tumor thrombi, suggesting a potential malignant phenotype.

The effect of VIP on bone metabolism has not been established, although it was reported that VIP receptors are expressed by both osteoblasts and osteoclasts [14, 15]. The present case has provided the first clinical evidence that VIPoma is associated with a dysfunction of bone metabolism, as exemplified by the increase of osteocalcin, P1NP and most significantly, beta-CTX (Table 1). After surgery, beta-CTX subsided, and P1NP rose significantly (Table 1), suggesting a shift from bone resorption toward bone formation. It is very interesting to note that another patient with VIPoma, who was diagnosed recently in our hospital, has shown a very similar pattern of changes in bone metabolism before and after surgery (Jiang et al., unpublished data). It has been reported that WDHA syndrome is often accompanied by hypercalcemia [13]. The current case showing a dysfunction of bone metabolism suggests a direct effect of VIP on osteoclasts, which should be considered as the underlying mechanism of hypercalcemia in the patients with VIPoma.

The most impressive finding of this case is the miraculous recovery of renal function after surgical treatment. To our knowledge, this is the first case of VIP-secreting pheochromocytoma with such a high level of Cr that hemodialysis was needed. In considering that the diarrhea and dehydration lasted for several months, pre-renal azotemia was suspected initially. As such, chances for recovery would be slim. But after surgical removal of the adrenal mass, serum Cr decreased dramatically together with a gradual increase in urine volumes, and dialysis was no longer needed. Another interesting finding in this case is that, despite extremely high level of NMN, the patient had no hypertensive symptoms. Such a typical characteristic of pheochromocytoma was probably masked by the vasodilative effect of VIP and severe dehydration due to diarrhea.

Because both NMN and calcitonin were elevated, multiple endocrine neoplasia type 2 (MEN2) was suspected in the initial diagnosis. Upon investigation, the patient had no relevant family history and the histology of thyroid specimen did not support the diagnosis of MEN2. It should also be mentioned that NMN was significantly elevated in the present case, whereas pheochromocytomas in MEN2 typically produce epinephrine, leading to elevated MN [16, 17]. Moreover, a fast screening of genomic DNA in our patient’s sample failed to identify any common RET gene mutations. We have also performed sequencing on the whole coding region of RET using cDNA from thyroid tissues of our patient, and no mutation was identified. Based on these investigations, along with the patient’s clinical manifestations, we could exclude the possibility of MEN2. So far, the only genetic manifestation of pheochromocytoma associated with WDHA syndrome was found being a *NF1* gene mutation, with two cases reported in the literature [8, 18]. A pheochromocytoma secreting calcitonin has been previously reported in a MEN2A patient bearing a triple RET gene mutation [19]. Yet the reason for the elevated calcitonin in this case remains unknow, as the IHC for calcitonin was negative in both pheochromocytoma and the thyroid nodule of the patient.

## Conclusions

In conclusion, the current report describes a very rare case of WDHA syndrome caused by a VIP-positive pheochromocytoma. It is also the first case of WDHA that progressed to uremia relying on hemodialysis, and yet recovered completely after surgical removal of the pheochromocytoma. In addition, this was also reported for the first time that a VIP-positive pheochromocytoma had significant effects on bone metabolism in the patient.

### Consent

Written informed consent was obtained from the patient for publication of this case report and any accompanying images. A copy of the written consent is available for review by the Editor of this journal.

## Abbreviations

WDHA: Watery diarrhea, hypokalemia and achlorhydria
VIP: Vasoactive intestinal polypeptide
IHC: Immunohistochemistry
PET-CT: Positron emission tomography-computed tomography
SUV: Standardized uptake value
PTH: Parathyroid hormone
P1NP: Procollagen type 1 aminoterminal Propeptide
beta-CTX: Cross-linked C-terminal telopeptide of type 1 collagen
MN: Metanephrine
NMN: Normetanephrine
MEN2: Multiple endocrine neoplasia type 2
NF-1: Neurofibromatosis type 1.

## References

[1] Verner JV, Morrison AB (1958). Islet cell tumor and a syndrome of refractory watery diarrhea and hypokalemia. Am J Med.

[2] Loehry CA, Kingham JG, Whorwell PJ (1975). Watery diarrhoea and hypokalaemia associated with a phaeochromocytoma. Postgrad Med J.

[3] Cooperman AM, Desantis D, Winkelman E, Farmer R, Eversman J, Said S (1978). Watery diarrhea syndrome. Two unusual cases and further evidence that VIP is a humoral mediator. Ann Surg.

[4] Fisher BM, MacPhee GJ, Davies DL, McPherson SG, Brown IL, Goldberg A (1987). A case of watery diarrhoea syndrome due to an adrenal phaeochromocytoma secreting vasoactive intestinal polypeptide with coincidental autoimmune thyroid disease. Acta Endocrinol (Copenh).

[5] Matta MK, Prorok JJ, Trimpi HD, Sheets JA, Stasik JJ, Khubchandani IT (1978). WDHA syndrome caused by pheochromocytoma: report of a case. Dis Colon Rectum.

[6] Ozbay A, Obukhau A, Buhl L, Brondt Hartlev L, Logstrup Poulsen P (2008). Adrenal pheochromocytoma producing vasoactive intestinal peptide and masking hypertension. Horm Res.

[7] Pais SO (1978). Angiographic demonstration of a vasoactive intestinal polypeptide-secreting pheochromocytoma in a patient with WDHA syndrome. AJR Am J Roentgenol.

[8] Quarles Van Ufford-Mannesse P, Castro Cabezas M, Vroom TM, Van Gils A, Lips CJ, Niermeijer P (1999). A patient with neurofibromatosis type 1 and watery diarrhoea syndrome due to a VIP-producing adrenal phaeochromocytoma. J Intern Med.

[9] Sackel SG, Manson JE, Harawi SJ, Burakoff R (1985). Watery diarrhea syndrome due to an adrenal pheochromocytoma secreting vasoactive intestinal polypeptide. Dig Dis Sci.

[10] Smith SL, Slappy AL, Fox TP, Scolapio JS (2002). Pheochromocytoma producing vasoactive intestinal peptide. Mayo Clin Proc.

[11] Van Eeckhout P, Shungu H, Descamps FX, Lanthier P, Castelain T, Saey JP, Rettman R, Drese C, Colin IM (1999). Acute watery diarrhea as the initial presenting feature of a pheochromocytoma in an 84-year-old female patient. Horm Res.

[12] Viale G, Dell’Orto P, Moro E, Cozzaglio L, Coggi G (1985). Vasoactive intestinal polypeptide-, somatostatin-, and calcitonin-producing adrenal pheochromocytoma associated with the watery diarrhea (WDHH) syndrome. First case report with immunohistochemical findings. Cancer.

[13] Metz DC, Jensen RT (2008). Gastrointestinal neuroendocrine tumors: pancreatic endocrine tumors. Gastroenterology.

[14] Lundberg P, Lie A, Bjurholm A, Lehenkari PP, Horton MA, Lerner UH, Ransjo M (2000). Vasoactive intestinal peptide regulates osteoclast activity via specific binding sites on both osteoclasts and osteoblasts. Bone.

[15] Lundberg P, Lerner UH (2002). Expression and regulatory role of receptors for vasoactive intestinal peptide in bone cells. Microsc Res Tech.

[16] Eisenhofer G, Lenders JW, Linehan WM, Walther MM, Goldstein DS, Keiser HR (1999). Plasma normetanephrine and metanephrine for detecting pheochromocytoma in von Hippel-Lindau disease and multiple endocrine neoplasia type 2. N Engl J Med.

[17] Eisenhofer G, Walther MM, Huynh TT, Li ST, Bornstein SR, Vortmeyer A, Mannelli M, Goldstein DS, Linehan WM, Lenders JW, Pacak K (2001). Pheochromocytomas in von Hippel-Lindau syndrome and multiple endocrine neoplasia type 2 display distinct biochemical and clinical phenotypes. J Clin Endocrinol Metab.

[18] Onozawa M, Fukuhara T, Minoguchi M, Takahata M, Yamamoto Y, Miyake T, Kanagawa K, Kanda M, Maekawa I (2005). Hypokalemic rhabdomyolysis due to WDHA syndrome caused by VIP-producing composite pheochromocytoma: a case in neurofibromatosis type 1. Jpn J Clin Oncol.

[19] Conzo G, Circelli L, Pasquali D, Sinisi A, Sabatino L, Accardo G, Renzullo A, Santini L, Salvatore F, Colantuoni V (2012). Lessons to be learned from the clinical management of a MEN 2A patient bearing a novel 634/640/700 mutation of the RET proto-oncogene. Clin Endocrinol.

[CR20] The pre-publication history for this paper can be accessed here:http://www.biomedcentral.com/1471-2407/14/553/prepub

